# Genome-Wide Identification of Circular RNAs Revealed the Dominant Intergenic Region Circularization Model in *Apostichopus japonicus*

**DOI:** 10.3389/fgene.2019.00603

**Published:** 2019-07-02

**Authors:** Xuelin Zhao, Xuemei Duan, Jianping Fu, Yina Shao, Weiwei Zhang, Ming Guo, Chenghua Li

**Affiliations:** ^1^State Key Laboratory for Quality and Safety of Agro-products, Ningbo University, Ningbo, China; ^2^Laboratory for Marine Fisheries Science and Food Production Processes, Qingdao National Laboratory for Marine Science and Technology, Qingdao, China

**Keywords:** *Apostichopus japonicus*, circRNA, intergenic region circularization, miRNA, skin ulceration syndrome

## Abstract

Circular RNAs (circRNAs) were recently recognized to act as competing endogenous RNAs and play roles in gene expression regulation. Previous studies in humans and silkworms have shown that circRNAs take part in immune regulation. Here, we conducted coelomocyte circRNA sequencing to explore its immune functions in healthy and skin ulceration syndrome (SUS)-diseased sea cucumbers. A total of 3,592 circRNAs were identified in libraries with diversified circularization patterns compared with animal models. The common intron-pairing-driven circularization models are not popular in sea cucumber genome, which was replaced with intergenic region circularization. The accuracy of these identified circRNAs was further validated by Sanger sequencing and RNase R-treated assays. Expression profile analysis indicated that 117 circRNAs were upregulated and 144 circRNAs were downregulated in SUS-diseased condition, of which 71.6% were intergenic-type circRNAs. The interaction network of differentially expressed circRNAs and microRNAs (miRNAs) was constructed and showed that miR-2008 and miR-31, detected with significantly differential expression in SUS-affected samples in a previous study, were predicted to be regulated by 10 and 11 differentially expressed circRNAs with more than 10 binding sites, respectively. Moreover, seven circRNAs were further validated by quantitative real-time PCR, whose variation trends were consistent with circRNA sequencing. All our results supported that intergenic-type circRNAs might have a dominant function in *Apostichopus japonicas* immune response by acting as miRNA regulators.

## Introduction

In the past decades, several non-coding RNAs were found to have genetic functions, including regulating protein coding gene expression from transcription to translation and regulating mature protein function (Hansen et al., 2016). RNAs were widely believed to have a linear structure; thus, circular RNAs (circRNAs) were identified as “exon shuffling” since their discovery more than 20 years ago (Nigro et al., 1991). Recently, RNAs have been found to exist in circular forms and possess biological functions (Burd et al., 2010). CircRNAs are generally formed by “back-splicing” of precursor messenger RNAs (mRNAs), in which an upstream 3′ splice acceptor is joined to a downstream splice donor (Barrett et al., 2015). Owing to its circular form, circRNAs are more stable than linear RNAs and can survive under RNase R treatment (Suzuki et al., 2006).

The breakthroughs in high-throughput sequencing and algorithms of data analysis have led to the discovery of circRNAs in humans (Memczak et al., 2013; Salzman et al., 2013; Rybak-Wolf et al., 2015), mices (Memczak et al., 2013), insects (Westholm et al., 2014; Gan et al., 2017), fish (Nitsche et al., 2013; Shen et al., 2016), and nematodes (Ivanov et al., 2015). These reports indicated that circRNAs are abundant and present in invertebrates and vertebrates. However, the mechanism of RNA circularization is not well understood. The flanking introns of circularized exons are long and contain complementary ALU repeats in mammals (Jeck et al., 2013). Previous studies suggested that circRNAs originate not only from exons but also from introns and intergenic regions. A recent study found that exon–intron circRNAs are predominantly localized in the nucleus (Li Z. et al., 2015).

In the species where circRNAs were discovered, some circRNAs acted as competing endogenous RNAs with microRNA response elements to regulate the expression of microRNAs (miRNAs), and these circRNAs were defined as “miRNA sponges” (Ebert et al., 2007). This function of circRNAs was first found in murine SRY with 16 binding sites of miR-138 and CDR1as with more than 70 binding sites of miR-7 (Hansen et al., 2013). Since then, several circRNAs that contain dozens of miRNA binding sites were also found in *Drosophila* (Westholm et al., 2014), zebrafish (Shen et al., 2016), and silkworm (Hu et al., 2018).

*Apostichopus japonicus* is an important aquaculture species in China. This species often suffers from skin ulceration syndrome (SUS). Many studies have been conducted to better understand the pathogenesis of SUS from miRNA, mRNA, and protein expression profiles (Li et al., 2012; Zhang P. et al., 2014; Gao Q. et al., 2015). Eight miRNAs that displayed differential expression were identified in coelomocyte between SUS-affected and healthy sea cucumbers (Li et al., 2012). CircRNAs exhibit significantly different expressions in diseased tissues (Bachmayr-Heyda et al., 2015). Some circRNAs play a key role as miRNA sponges in human diseases (Burd et al., 2010; Caiment et al., 2015; Li F. et al., 2015) and viral infections (Hu et al., 2018). However, their functions in sea cucumbers suffering from SUS remains unknown. Thanks to the release of genome of *A. japonicus*, more and more studies could be conducted. There are 30,350 protein-coding genes distributing in 22 chromosomes with an assembly of approximately 805 Mb in the genome of *A. japonicus* (Zhang et al., 2017), which is essential for the identification of circRNAs.

*A. japonicus* has an important evolutionary status. Therefore, its circularization patterns were valuable to explore evolutionary process from invertebrates to mammals. In addition, we performed deep transcriptome sequencing of coelomocyte from healthy and SUS-diseased sea cucumbers to identify the circRNAs and illuminate the potential functions of gene-encoded circRNAs under SUS challenges. The results of this study could increase the circRNA database and enrich circRNA function.

## Materials and Methods

### Sample Collection

Thirty adult sea cucumbers (120 ± 5 g) with SUS were collected from Pulandian Hatchery in Dalian, China in April 2017. The same number of healthy samples with the same raising condition were also used as controls. The coelomic fluids (filled in the large and extensive body cavities in most echinoderms) were collected from five individuals as a sample and centrifuged at 800 × *g* at 4°C for 5 min to harvest coelomocytes (populations of free cells in coelomic fluid). The samples were then stored at −80°C until use.

### RNA Extraction and Library Preparation

Total RNAs were extracted using TRIzol (Life Technologies) according to the manufacturer’s instructions. RNA integrity was checked by agarose gel electrophoresis. The quality and quantity of total RNA were evaluated using the Agilent 2100 Bioanalyzer (Agilent Technologies).

Approximately 5 μg of total RNA per sample was used for library preparation and sequencing according to the standard library construction protocol. Briefly, ribosomal RNAs were depleted using Epicentre Ribo-Zero^TM^ rRNA Removal Kit (Epicentre) and purified by ethanol precipitation. Afterward, the purified RNAs were used to construct the sequencing libraries with the NEBNext^®^ Ultra^TM^ Directional RNA Library Prep Kit for Illumina^®^ (NEB). Finally, the libraries were sequenced on an Illumina HiSeq X Ten platform with 150-bp paired-end reads.

### Prediction of CircRNAs

To ensure the abundance and accuracy of identified circRNAs, three pipelines were used for circRNA identification, including find_circ (Memczak et al., 2013), circRNA_finder (Westholm et al., 2014), and CIRI (Gao Y. et al., 2015). On the basis of the recently released genome sequence of sea cucumber (Zhang et al., 2017), the find_circ pipeline started by removing reads that could be aligned to the genome with the full length. The remaining reads were analyzed using find_circ with the following parameters: find_circ.py unmapped_anchors.sam –G haishen20161129.fasta –p prefix –s find_circ.sites.log –R find_circ.sites.reads > find_circ.sites.bed 2; grep CIRCULAR find_circ.sites.bed | grep –v chrM | gawk “$5>=2” | grep UNAMBIGUOUS_BP | grep ANCHOR_UNIQUE | python maxlength.py 100000 > find_circ.candidates.bed. CircRNA identification using circRNA_finder was based on the results of the STAR read aligner with the default parameters: runStar.pl R1.fastq R2.fastq (STAR genome) circRNA/prefix; postProcessStarAlignment.pl circRNA/prefix prefix. The default parameters of CIRI version 2.0.2 were: CIRI_v2.0.2.pl –I input.sam –O output_circRNAs.txt –F haishen20161129.fasta –A CIRI.candidates.gtf. Finally, the reads that were predicted in each pipeline and appeared at least twice remained.

### Analysis of CircRNA Features

CircRNAs were divided into four types using CircView (Feng et al., 2017), including exonic, exon–intronic, intronic, and intergenic circRNAs. For the exonic and exon–intronic types of circRNAs, we counted the number of exons in circRNAs. The flanking sequences of the above two types of circRNAs that contained the nearest intron sequences were analyzed by Blastn (*E*-value <1e^−5^) to determine reverse complementary sequences. Moreover, the flanking sequences of intronic and intergenic circRNAs were analyzed in the range of 200 nucleotides. The circRNA homologs in sea cucumber were compared with human, mouse, and nematode homologs using Blastn (*E*-value <1e^−5^). The human, mouse, and nematode circRNA sequences were downloaded from circBase^1^.

### Data Analysis

Expression levels of circRNAs were quantified using the number of junction spanning reads. The values were normalized to the total number of reads in the library using the following formula: [(junction spanning reads × 1,000,000)/total raw reads]. Differential expression analysis of the two samples was performed using edgeR (version 3.8.6) (Robinson et al., 2010) with FDR threshold <0.01 and |log_2_(fold_change)| >1.

The parent genes of circRNAs were annotated by Gene Ontology (GO) functional significance and Kyoto Encyclopedia of Genes and Genomes (KEGG) pathway analyses. The GO functional annotation and enrichment analyses were conducted by using TBtools (Chen et al., 2018) for assigning GO terms, including the domains of biological process (BP), cellular component (CC), and molecular function (MF). We also performed KEGG pathway analysis to harvest pathway clusters covering the molecular interaction and reaction networks of the parent circRNA genes by using the KOBAS software (Xie et al., 2011). In addition, the top 20 enriched pathways were selected on the basis of the *p*-value and enrichment score.

For miRNA site analysis, miRanda software was used to predict the miRNA binding sites in circRNAs with the default parameters (Enright et al., 2003). The miRNAs used in this study were the conserved miRNAs of sea cucumbers reported in a previous study (Li et al., 2012). We selected and analyzed the target circRNAs with a free energy threshold of 15 kcal/mol from the original predictions. A circRNA–miRNA interaction network was constructed in accordance with the number of miRNA sites in the differentially expressed circRNAs and visualized by using Cytoscape (version 3.6.1) (Shannon et al., 2003).

### PCR and Quantitative Real-Time PCR (qRT-PCR) Validation

To validate the reliability of circRNA, two circRNAs were randomly selected from each type of circRNA, including exon–exonic, exon–intronic, intronic, and intergenic circRNAs with divergent primers (Supplementary Table S1), which were designed to amplify the splicing junction of circRNAs. The total RNAs were reversed to cDNA with the random primer using a PrimeScript RT reagent Kit with gDNA Eraser (Takara) according to the manufacturer’s instructions. The PCR parameters were as follows: 94°C for 3 min, followed by 40 cycles at 94°C for 30 s, an appropriate annealing temperature (according to the melting temperature of the primers) for 45 s and 72°C for 30 s, and 72°C for 2 min at the end. The products were confirmed by Sanger sequencing.

For RNase R digested samples, 4 μg of total RNA and 1 μL of RNase R (Epicentre 10 U/μg) were incubated at 37°C for 15 min. After the reaction, treated RNA was purified with acidic P/C/I and ethanol precipitated. The samples were used to test the integrity of circRNAs and compared with the corresponding linear mRNA after RNase R treatment.

In addition, to validate the expression of circRNAs analyzed by CIRI, seven circRNAs were randomly selected and validated by quantitative RT-PCR (five of them were also validated by Sanger sequencing) (Supplementary Table S1). This experiment was conducted with the ABI 7500 Real Time PCR System using TB Green Premix Ex Taq II (Takara) according to the manufacturer’s instruction. The expression levels of circRNAs were normalized to β-actin.

## Results

### Genomic Features of *A. japonicus* CircRNAs

**FIGURE 1:**
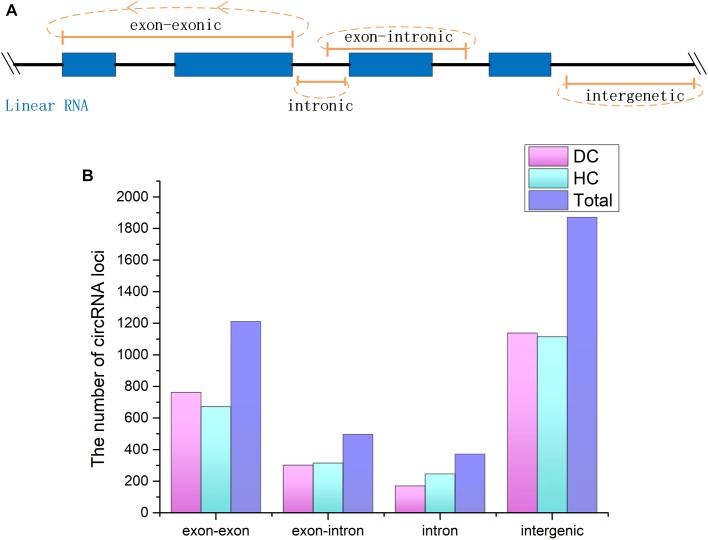
CircRNAs identified from *Apostichopus japonicas* coelomocyte. **(A)** Four types of circRNAs according to their genomic location. **(B)** Distribution of the number of circRNAs from different types in DC, HC groups and in total.

CircRNAs are a newfound category of non-coding RNAs without the 3′ ploy (A) tail, which can be lost during the general enrichment procedure of mRNAs. However, more informative parts of the transcriptome can be enriched and sequenced by removing rRNA from the total RNA. In total, 211,004,636 and 236,349,396 raw reads with 150 bp in length were generated from coelomocytes of diseased (DC group) and healthy (HC group) sea cucumbers, respectively, and 207,620,356 and 231,127,910 clean reads were obtained accordingly after removing adapter sequences and low-quality reads (Supplementary Table S2). All raw data have been submitted to the NCBI SRA database (BioProject ID PRJNA512578). According to the prediction results, a total of 3,952 circRNAs were identified. Among them, 455 circRNAs were commonly predicted by all three algorithms, including find_circ, CIRI, and circ_finder. On the basis of the genomic location information, four types of circRNAs were classified, including exon–exonic, exon–intronic, intronic, and intergenic, which comprised 30.5, 12.7, 9.4, and 47.3%, respectively. Among the expressed circRNAs in DC and HC groups, intergenic circRNAs are also the largest category (Figure 1 and Supplementary Table S3).

**FIGURE 2:**
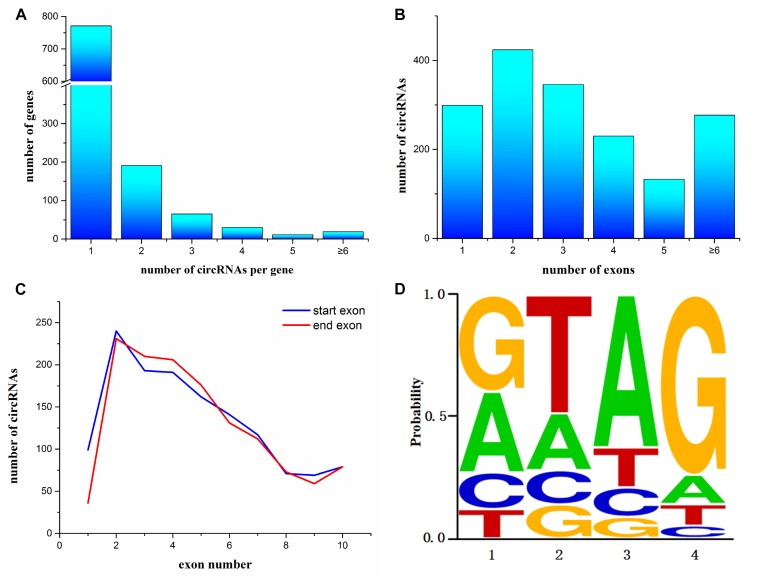
Characterization of circularization in *Apostichopus japonicas*. **(A)** Distribution of circRNAs among genes. **(B)** Number of exons contained within exonic circRNAs. **(C)** Start and end exons of circRNAs. CircRNAs are most enriched for exons involving positions 2–4. **(D)** Splice signals of identified circRNAs.

To annotate the *A. japonicus* circRNAs within exons to the reference genome, we found that 1,087 genes express at least one circRNA. Among them, most genes (771 genes) expressed a single circRNA, but at the same time, some genes were found to generate a large number of circRNAs (Figure 2A). For example, AJAP11965 produced 24 different circRNAs, which is the maximum quantity. In addition, we observed the number of exons within one circRNA (Figure 2B), which ranged from 1 to 27. Supplementary Figure S1 shows some examples, including single-exon circularization event, a few exons contained in one circRNAs, and even alternative and interleaved events, where the same splice sites can take part in more than one splicing reactions. However, circRNAs containing two exons were the most common form (Figure 2B). Moreover, the start and end exons of circRNAs were mostly enriched at position 2 of gene models. For the end exon, positions 3 and 4 were significant as well (Figure 2C). This finding indicates that circularization was biased to the 5′ end of genes.

Many circRNAs found in humans have long introns flanking with complementary ALU repeats, which is the precondition of the intron-pairing-driven circularization model (Jeck et al., 2013). The flanking intron sequences of circRNA in *A. japonicus* were analyzed to investigate the probable motif in circRNA biogenesis. Unlike the results obtained from humans, only 321 circRNAs were identified with reverse complementary flanking sequences from a total of 3,952 circRNAs, which were found in all types of circRNAs. However, the number of pairing in each type of circRNAs cannot illustrate the correlation. On the basis of the classification, 167 exonic circRNAs (including exonic and exon–intronic types of circRNAs), 51 intronic circRNAs, and 203 intergenic circRNAs were found. This finding indicated that only 9.8% (167/1709) of exonic circRNAs of sea cucumber meet the requirement of intron-pairing-driven circularization model. Moreover, the splicing signals of the circRNAs identified from *A. japonicus* were investigated, and most of them had the canonical GT/AG signal (Figure 2D).

To assess the conservation of circRNAs between different species, we compared the sea cucumber circRNAs with human, mouse, and *Caenorhabditis elegans* circRNAs from circBase. *A. japonicus* circRNAs were slightly homologous with other species. In total, 92 circRNAs were homologous with humans, 44 circRNAs were homologous with mouse, and only one circRNA was homologous with *C. elegans* out of 724 nematode circRNAs. Of the 44 circRNAs that were homologous with mouse, 36 circRNAs were the same as the homolog of human. All Blast results are listed in Supplementary Table S4.

### Functional Annotation of Parental Genes

**FIGURE 3:**
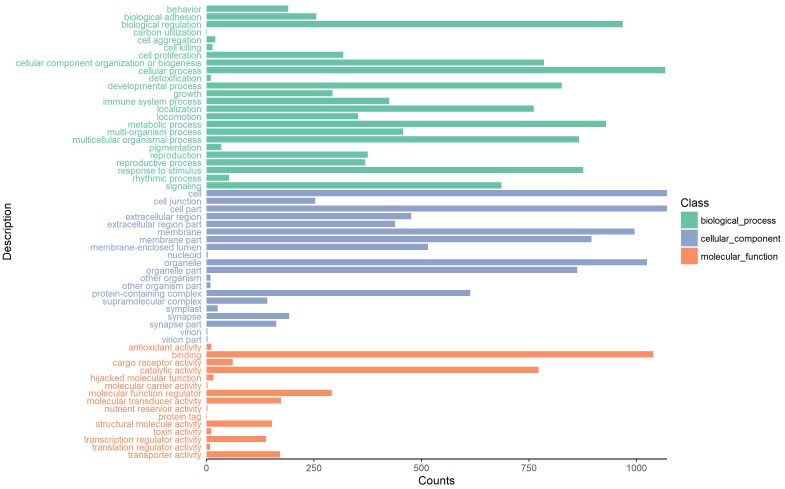
GO analysis of the host genes of circRNAs under the categories of BP, CC, and MF.

**FIGURE 4:**
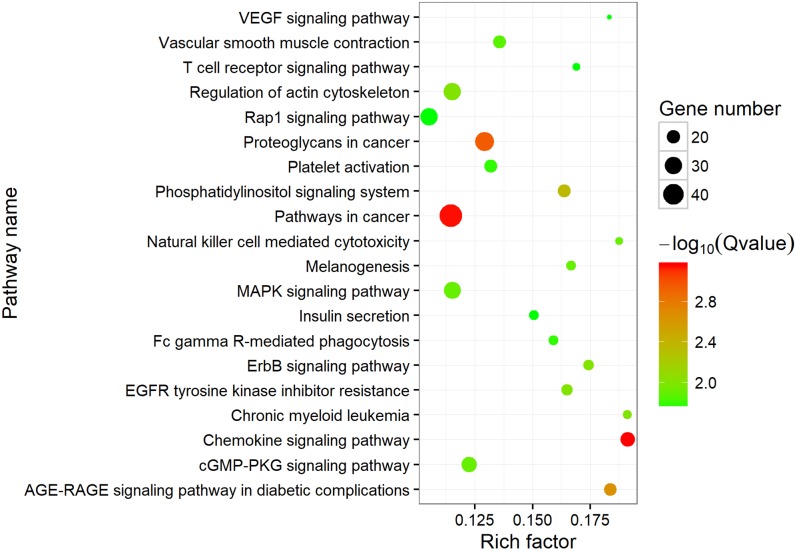
Top 20 enriched KEGG pathways. The size of the circle represents the number of genes. Green to red indicates that the corrected *p*-value is gradually becoming smaller.

To investigate the putative function of circRNAs, the annotation of their corresponding linear genes is a common strategy. This procedure is based on the hypothesis some inner functional interaction between circRNAs and their parental genes exists. In this study, we conducted GO and KEGG annotations to explore the response of sea cucumber circRNAs under SUS. Excluding intergenic circRNAs, 1,122 parental genes with GO annotation were classified into three GO categories (Figure 3). For BP, the top three subcategories were cellular process (1069), biological regulation (968), and metabolic process (929), followed by response to stimulus (876). For CC, cell (1071), cell part (1071), and organelle (1024) were the three largest groups. Binding (1039), catalytic activity (772), and molecular function regulator (291) were the top three subcategories in MF. A total of 819 parental genes were annotated to 276 pathways. Against the background of all genes in the *A. japonicus* genome, the KEGG enrichment analysis showed that the significantly enriched pathways of the parental genes were chemokine signaling pathway (KO04062), pathways in cancer (KO05200), and proteoglycans in cancer (KO05205) (Figure 4). Furthermore, nearly half of the top 20 enriched pathways were related to signaling pathways (Figure 4).

### Differentially Expressed CircRNAs Under SUS

The expression profiles of circRNAs from coelomocytes in diseased and healthy sea cucumbers were measured by mapped back-splicing junction reads. A total of 2,464 circRNAs were expressed in both libraries, whereas 756 and 732 circRNAs were expressed specifically in the diseased and healthy samples, respectively. A total of 261 differentially expressed circRNAs were filtered by |log_2_(fold_change)| >1 and FDR <0.01, which included 117 upregulated and 144 downregulated circRNAs in the coelomocytes of SUS-infected sea cucumbers (Supplementary Figure S2 and Supplementary Table S5). According to the types of circRNAs, the differentially expressed circRNAs could be classified into 20 intronic, 54 exonic (including exon–exonic and exon–intronic), and 187 intergenic circRNAs. Seventy-four differentially expressed circRNAs were from 40 liner genes, which were conducted by GO enrichment analysis regarding all the parental genes of circRNAs we found as background. The enriched terms with *p*-value less than 0.05 were selected and ranked by their *p*-values according to the routine GO classification algorithms. For the dysregulated circRNAs, the top three GO processes included cell–cell adhesion, cell adhesion, and cell activation in the BP subgroup. Fibrinogen complex, blood microparticle, and phagocytic cup were the top three processes in the CC subgroup, whereas G-protein coupled receptor activity, transmembrane signaling receptor activity, and signaling receptor activity were the top three processes in the MF subgroup (Supplementary Table S6).

### CircRNA and miRNA Interaction Network

**FIGURE 5:**
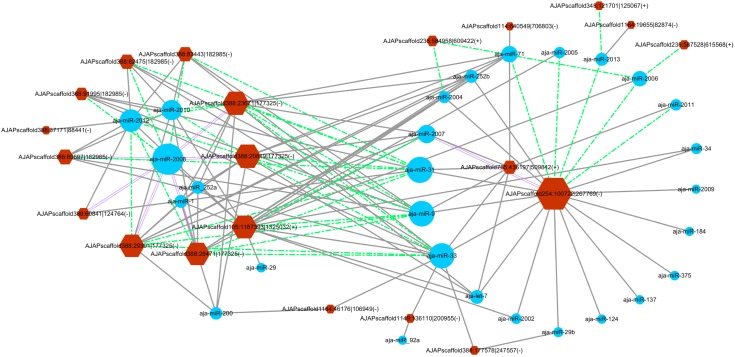
Differentially expressed circRNA–miRNA interaction network in the sea cucumber under SUS challenges. Hexagon nodes represent circRNAs, and circle nodes represent miRNAs. The size of the node represents the number of interacted relationships. Gray lines represent more than 10 miRNA binding sites in the circRNA. Green dashed lines represent more than 20 miRNA binding sites in the circRNA. Purple double lines represent more than 30 miRNA binding sites in the circRNA.

The miRanda algorithm was used to detect the targeted relationship between circRNAs and miRNAs in *A. japonicus*. The results showed that 3,679 out of 3,952 circRNAs had miRNA binding sites with high binding force. The number of binding sits for one miRNA on the circRNAs were listed in Supplementary Table S7. AJAPscaffold156:471337| 584617 had 622 miRNA binding sites, which is the circRNA with the greatest number of miRNA binding sites. On the basis of the results, many circRNAs can bind with more than one miRNA and have several binding sites targeted to the same miRNA. To determine the regulated function of the differentially expressed exonic circRNAs, 54 exonic circRNAs with more than 10 miRNA binding sites targeted to the same miRNA are displayed in Figure 5, which shows the interaction network of 21 circRNAs and 28 miRNAs. AJAPsacffold254:100722| 267769 had the largest number of miRNA sites with 20 miRNAs, whereas miR-2008 had the largest number of binding sites from circRNAs, followed by miR-31, miR-33, and miR-9. Ten differentially expressed circRNAs were derived from the same scaffold AJAPscaffold388 due to alternative splicing (Figure 5) with different expression trends. Each of them had more than 20 binding sites of miR-2008 and miR-31. This scaffold may be a good example for investigating the alternative splicing in circRNAs.

**FIGURE 6:**
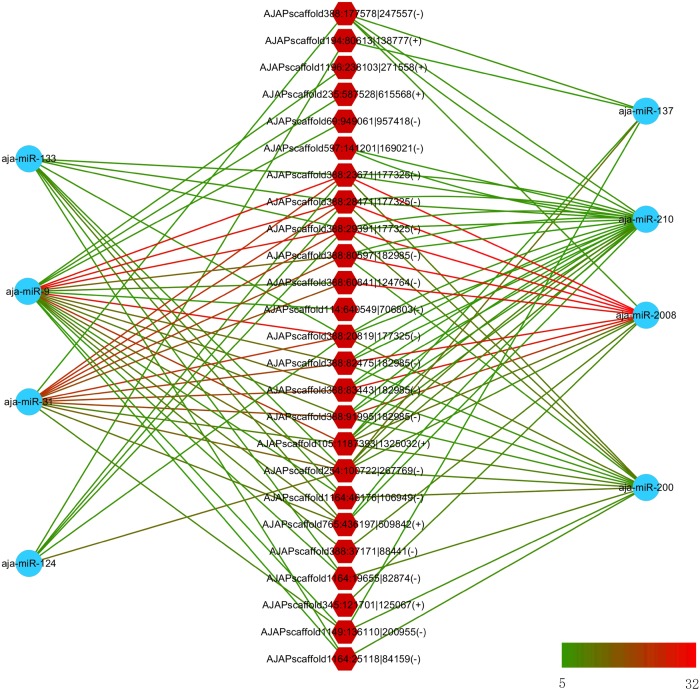
Interaction network between differentially expressed circRNAs and differentially expressed miRNA in sea cucumber under SUS challenges. Hexagon nodes represent circRNAs, and circle nodes represent miRNAs. Green to red line color represents the number of miRNA binding sites in the circRNA from 5 to 32.

In a previous study, eight miRNAs (miRNA-31, miRNA-2008, miRNA-210, miR-200, miR-133, miR-137, miR-9, and miR-124) were found to take part in the immune response under SUS (Li et al., 2012). We predicted the interaction force between the differentially expressed circRNAs and these eight miRNAs. Figure 6 shows the number of miRNA binding sites from 5 to 32, and miRNA-2008, miR-31, and miR-9 were the most regulated miRNAs by circRNAs.

### Experimental Verification of *A. japonicus* CircRNAs

**FIGURE 7:**
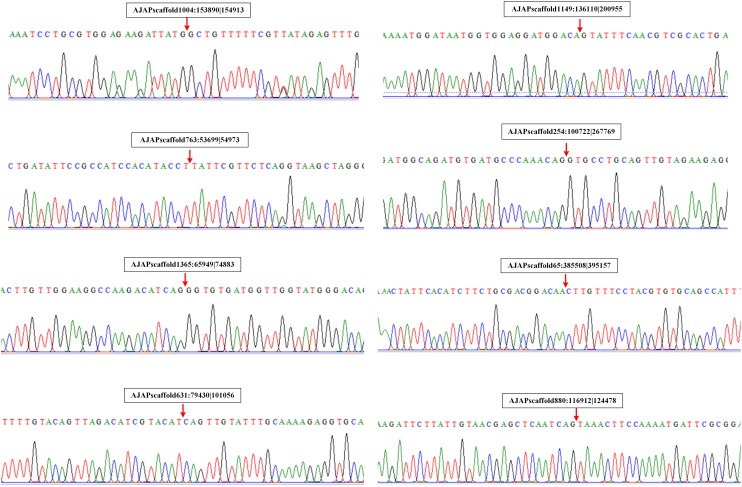
Validation of circRNAs by RT-PCR and Sanger sequencing. Red arrows represent back-splicing sites.

We also performed experimental validation of circRNAs in accordance with the transcriptomic sequencing results. Two circRNAs from each type were selected randomly using the divergent primers that should only amplify a back-spliced circRNA. The product of the expected size was confirmed by Sanger sequencing. The results showed that the sequences of the eight selected circRNAs were all identified with the 5′ and 3′ end of genome sequence covalently closed (Figure 7).

**FIGURE 8:**
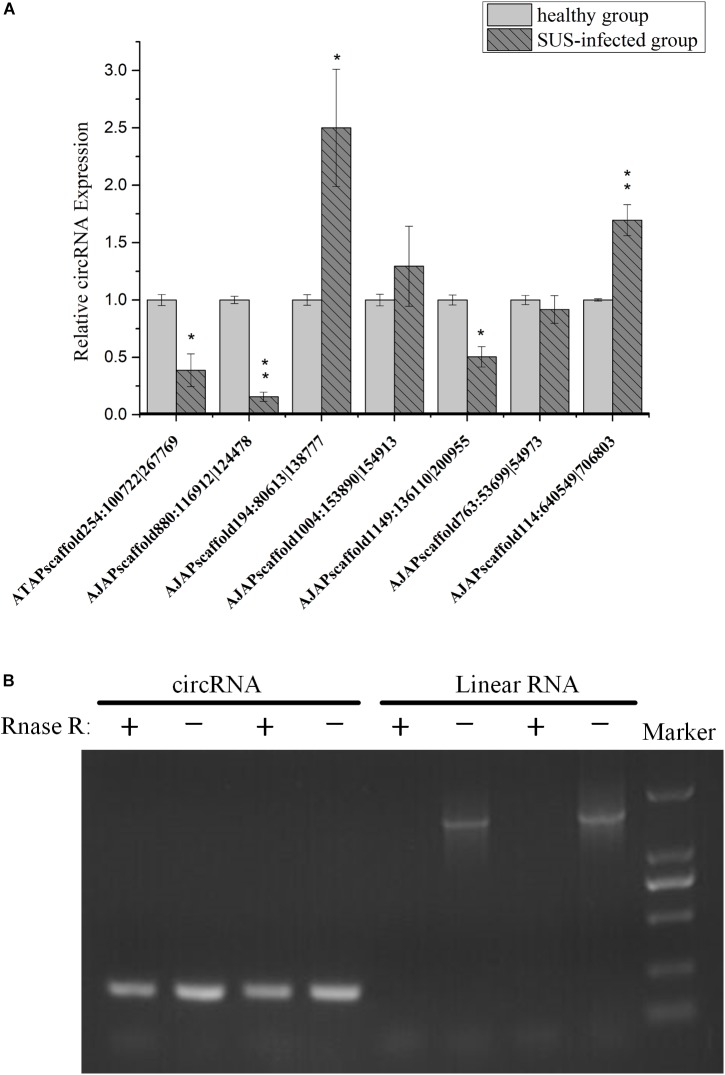
Experimental validation of circRNAs. **(A)** qRT-PCR analysis of seven circRNAs with different expression patterns in response to SUS. β-actin is the internal reference. *N* = 3, ^∗^*p* < 0.05, ^∗∗^*p* < 0.01. **(B)** Outward-facing primers and inward-facing primers were designed to compare total and RNase R-treated RNAs from coelomocytes by gel electrophoresis. Linear mRNA forms are depleted by RNase R, whereas circular ones are resistant. CircRNA: AJAPscaffold1149:136110| 200955; linear RNA: ficolin.

To understand the expression level change in coelomocyte in response to SUS, the expression pattern of seven circRNAs was investigated. The results showed that the expression tendencies were consistent with the analysis data, but qRT-PCR validation for most circRNAs showed fold changes less than those detected by RNA-seq differential expression analysis (Figure 8A). This high degree of discrimination was also found in other species possibly because qRT-PCR can amplify partially degraded transcripts (Westholm et al., 2014).

We also performed PCR tests of control and RNase R-treated samples using inward-facing primer sets, which amplify mRNA ficolin, and outward-facing primer sets, which amplify circRNA AJAPscaffold1149:136110| 200955, the host gene of which is ficolin according to annotation. We confirmed long full-length mRNAs and short circular transcripts on two samples. The expression of linear RNAs (ficolin) was diminished by RNase R treatment, whereas the circRNAs were slightly affected by RNase R treatment in the two samples (Figure 8B). CircRNAs could also be degraded with long-term RNase R incubation (Zhang Y. et al., 2016). We also found that different circRNAs can have different sensitivities to RNase R in the same incubation time (data not provided).

## Discussion

Skin ulceration syndrome is a sea cucumber-specific disease, which usually occurs in the summer and brings great economic loss in *A. japonicus* culture industries. The mRNA and miRNA expression profiles after SUS have been investigated in previous studies (Li et al., 2012; Yang et al., 2016). However, circRNA, a category of newly discovered RNA, has been identified in various cell lines and some model species. How they are transcribed in sea cucumbers and whether their expressions are affected by SUS remain unknown. In this study, we identified and analyzed the circRNA features and compared the circRNA expression profiles of coelomocytes of healthy and diseased sea cucumbers. In total, 3,952 circRNAs were found using three algorithms, considering the advantages and disadvantages between the algorithms observed and reliable predictions achieved by combining several algorithms in a previous study (Hansen et al., 2016). All the circRNAs can be shown by visualization tools to see their locations and expressions intuitively (Supplementary Figure S3). The comparison of circRNA features among different species indicated general characteristics and differences. For circRNAs comprising exons, circularization was biased to start from second exons of protein-coding genes and end in the second to fourth exons regardless of how many exons are there in gene models. This phenomenon was also observed in *Drosophila*, which showed high expressions of these circRNAs and was called the 5′ positional bias (Westholm et al., 2014). Canonical GT/AG splicing signals flanked in most circRNAs of sea cucumbers were also found to reflect back-splicing similar to other species. Furthermore, a single gene locus can produce multiple circRNAs through alternative back-splice site selection (Zhang X.-O. et al., 2016), and the alternative splicing events have been validated by experiments in human cell and fruit fly samples (Gao et al., 2016). The observation of alternative splicing events in sea cucumbers indicated that such splicing methods are conserved among different species. However, one circularization model certified that downstream donor and upstream acceptor could be promoted by the reverse complement sequences of the two flanking introns in humans (Jeck et al., 2013; Zhang X.-O. et al., 2014) and nematodes (Liu and Chen, 2015). No bias for the complementarity of flanking intronic sequences or flanking sequences of the circRNAs in sea cucumber was observed, even in *Drosophila* (Westholm et al., 2014) and zebrafish (Shen et al., 2016). In addition to this circularization model, the RNA binding protein Mbl promotes circularization in *Drosophila*, and alternative splicing may also participate in the formation of circRNAs (Ashwal-Fluss et al., 2014). Therefore, the circularization mechanism in sea cucumbers may be regulated by RNA binding proteins or other specific mechanisms not yet found, different from those of mammals.

CircRNAs were also found to be highly evolutionarily conserved in mammals (Salzman et al., 2012; Jeck et al., 2013). The proportion of zebrafish circRNAs that were homologous with humans and mice was more than 30% (Shen et al., 2016). Surprisingly, the circRNAs in sea cucumbers showed extremely low homology compared with those in humans, mice, and nematodes (less than 2%). On the one hand, this finding might have resulted from distant phylogenetic relationships. On the other hand, we found that the intergenic type of circRNAs in sea cucumbers can be aligned to the exon-type circRNAs in humans. This finding may indicate that some genes in humans were the pseudogene in *A. japonicus*, which suggested that the pseudogene might be one of the origins of circRNAs. We also tried to predict the putative function of circRNAs by annotating their host genes. The results of KEGG enrichment analysis showed that the host genes were highly enriched for the signaling pathways in coelomocytes. This finding suggested that circRNAs might play roles in signaling transduction in the immune system of sea cucumbers.

CircRNAs were found to take part in the regulation of diseases and pathogen infections in several organisms (Han et al., 2017; Hu et al., 2018; Tagawa et al., 2018). Furthermore, exploring the function of circRNAs in response to SUS in sea cucumbers, which are an important marine invertebrate both in aquaculture and evolution, is interesting. In the present study, 261 differentially expressed circRNAs were identified in SUS-diseased samples. According to the volcanic plot, the differentially expressed circRNAs dispersed dramatically from the steadily expressed ones. However, most circRNAs had lower abundance compared with linear RNAs without RNase R treatment. The expression of circRNAs with low abundance was also difficult to accurately quantify given the limited number of junction reads. Therefore, the selected parameters were strictly set with FDR less than 0.01 to ensure the differentially expressed circRNAs that were filtered are accurate. Moreover, GO enrichment analysis on host genes of these circRNAs was conducted to investigate the potential biological functions and mechanisms of the sea cucumber circRNAs in response to SUS disease. We found that cell adhesion and signaling receptor activities were enriched in GO categories. This finding indicated that circRNAs of coelomocytes mainly take part in the regulation of cell communication and signal receptors in response to SUS due to a clear bias for particular GO categories.

Perhaps the most impressive circRNA is CDR1as, which encodes a miRNA sponge (Hansen et al., 2013; Memczak et al., 2013), but this seems to be an exception. Some studies reported that the majority of circRNAs cannot function as miRNA sponges (Guo et al., 2014; Zhang X.-O. et al., 2014). However, some circRNAs, such as sex-determining region Y, circHIPK3, and cirITCH, contain many miRNA binding sites that can function as sponges (Capel et al., 1993; Li F. et al., 2015; Zheng et al., 2016). In *Drosophila*, the 5′ UTR and coding regions are the dominant exons involved in circRNAs and harbor thousands of well-conserved miRNA binding sites (Westholm et al., 2014). Therefore, miRNA sponges are still one of the important potential functions of circRNAs. In previous studies, we found that the expression patterns of many mRNAs and miRNAs changed under SUS challenges. In particular, eight miRNAs were identified as differentially expressed miRNAs in response to SUS (Li et al., 2012), and several miRNAs have been verified to modulate immune response via regulating their corresponding target genes (Lu et al., 2015a,b; Zhang et al., 2015). CircRNA–miRNA interaction networks were established in this study to predict the relationship of expressed exonic circRNAs and miRNA of sea cucumbers. CircRNAs with at least 10 miRNA binding sites of one miRNA and the relationship with their corresponding miRNAs are shown in Figure 5. Interestingly, miR-2008 and miR-31, detected with significantly differential expression in SUS-affected samples, were also predicted to be the most regulated miRNAs by 10 and 11 differentially expressed circRNAs, respectively. Several differentially expressed circRNAs had approximately 30 binding sites of miR-2008 and more than 20 binding sites of miR-31. This finding suggested that circRNAs might be the main upstream regulatory elements of miRNAs in response to immune defense under SUS challenges.

Future works can use the current results to delineate the immune function of individual circRNAs as miRNA sponges and uncover the mechanisms of RNA circularization during infection processes. The intergenic region circularization model needs more evidence to elucidate the bias mechanism. In addition, some circRNAs have marked differences in number and even no expression in healthy or affected groups. As a result, circRNAs may serve as a class of biomarkers for early warning of disease.

## Data Availability

The datasets for this manuscript are not publicly available because the circRNA sequencing data will be released after the paper is published. Requests to access the datasets should be directed to lichenghua@nbu.edu.cn.

## Ethics Statement

The sea cucumbers (*A. japonicus*) used in this study were commercially cultured animals, and all experiments were conducted in accordance with the recommendations in the Guide for the Care and Use of Laboratory Animals of the National Institutes of Health. The study protocol was approved by the Experimental Animal Ethics Committee of Ningbo University, China.

## Author Contributions

CL and XZ conceived and designed the experiments and wrote the manuscript. XZ, JF, and XD conducted the experiments. YS, MG, CL, and XZ analyzed the data. CL and WZ contributed to the reagents, materials, and analysis tools.

## Conflict of Interest Statement

The authors declare that the research was conducted in the absence of any commercial or financial relationships that could be construed as a potential conflict of interest.

## Footnotes

1 http://www.circbase.org

## Supplementary Material

FIGURE S1 Examples that illustrate the diversity in configurations of circularizing exons. **(A)** Single exon circularized. **(B)** Multiple exons in circle. **(C)** Complex and alternative circularization patterns.

FIGURE S2 Visualization of differentially expressed circRNAs induced by SUS.

FIGURE S3 Example of visualization of circRNAs on the genome by IGV. DC, HC: the expression of circRNAs in each group. genome_annotation.gff3: the annotation file of sea cucumber genome.

TABLE S1 Primers for validation of circRNAs.

TABLE S2 Statistics of the quality of circ-seq data.

TABLE S3 The genomic information and raw counts of circRNAs of *Apostichopus japonicus*.

TABLE S4 Blast results of *Apostichopus japonicus* compared with *Homo sapiens*, *Mus musculus*, and *Caenorhabditis elegans*.

TABLE S5 Differential expression analysis between SUS-deseased and healthy sea cucumbers.

TABLE S6 List of GO terms enriched in differentially expressed circRNAs.

TABLE S7 The number of binding sites between a circRNA-miRNA pair.
