# Supplementary figures and images for: Genome-Wide Identification of Circular RNAs Revealed the Dominant Intergenic Region Circularization Model in Apostichopus japonicus
Source: Front Genet. 2019 Jul 2;10:603. doi: 10.3389/fgene.2019.00603 (PMC6614181; doi:10.3389/fgene.2019.00603)

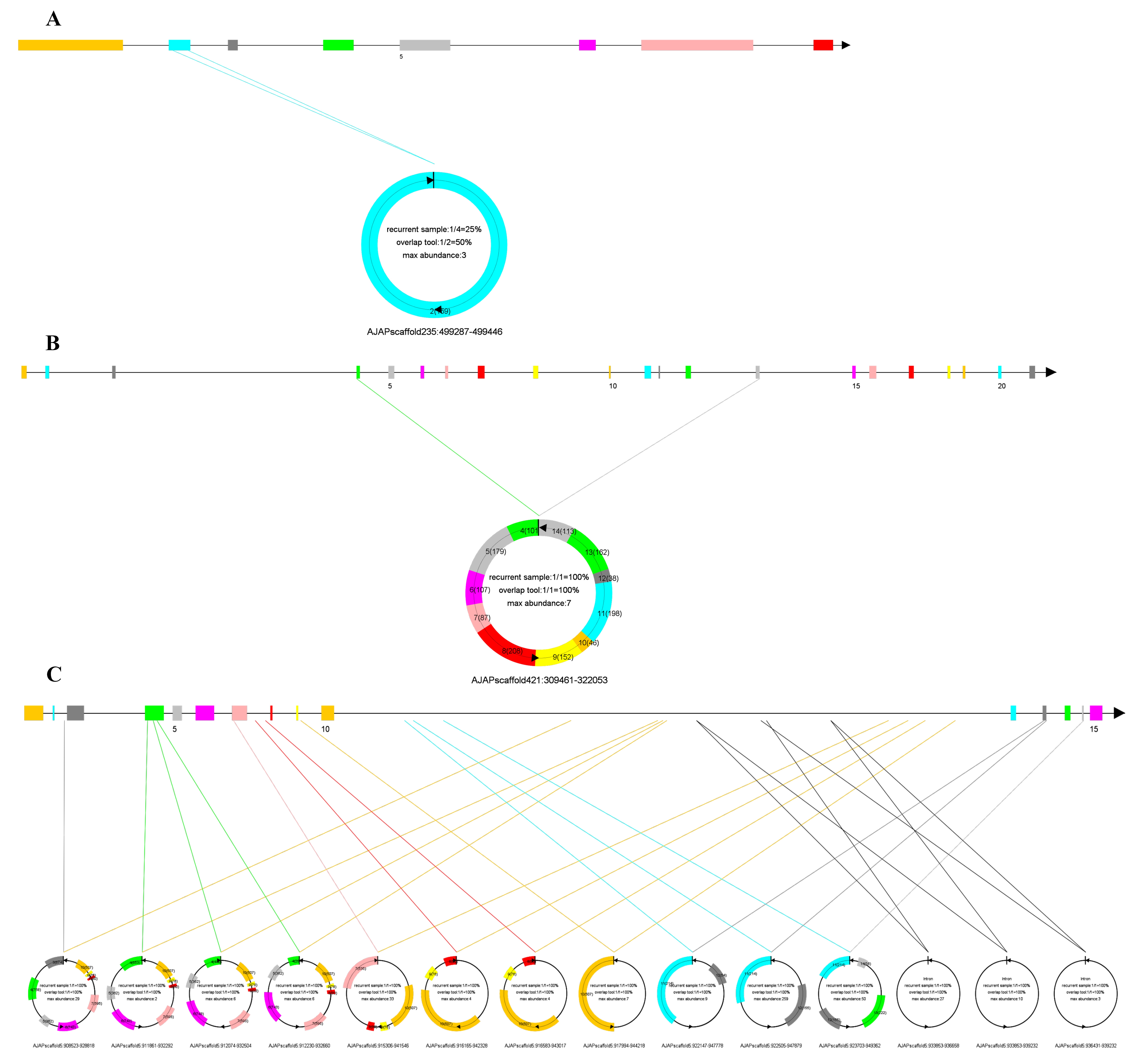

Supplement: FIGURE S1 — Examples that illustrate the diversity in configurations of circularizing exons. (A) Single exon circularized. (B) Multiple exons in circle. (C) Complex and alternative circularization patterns. [file Image_1.JPEG]

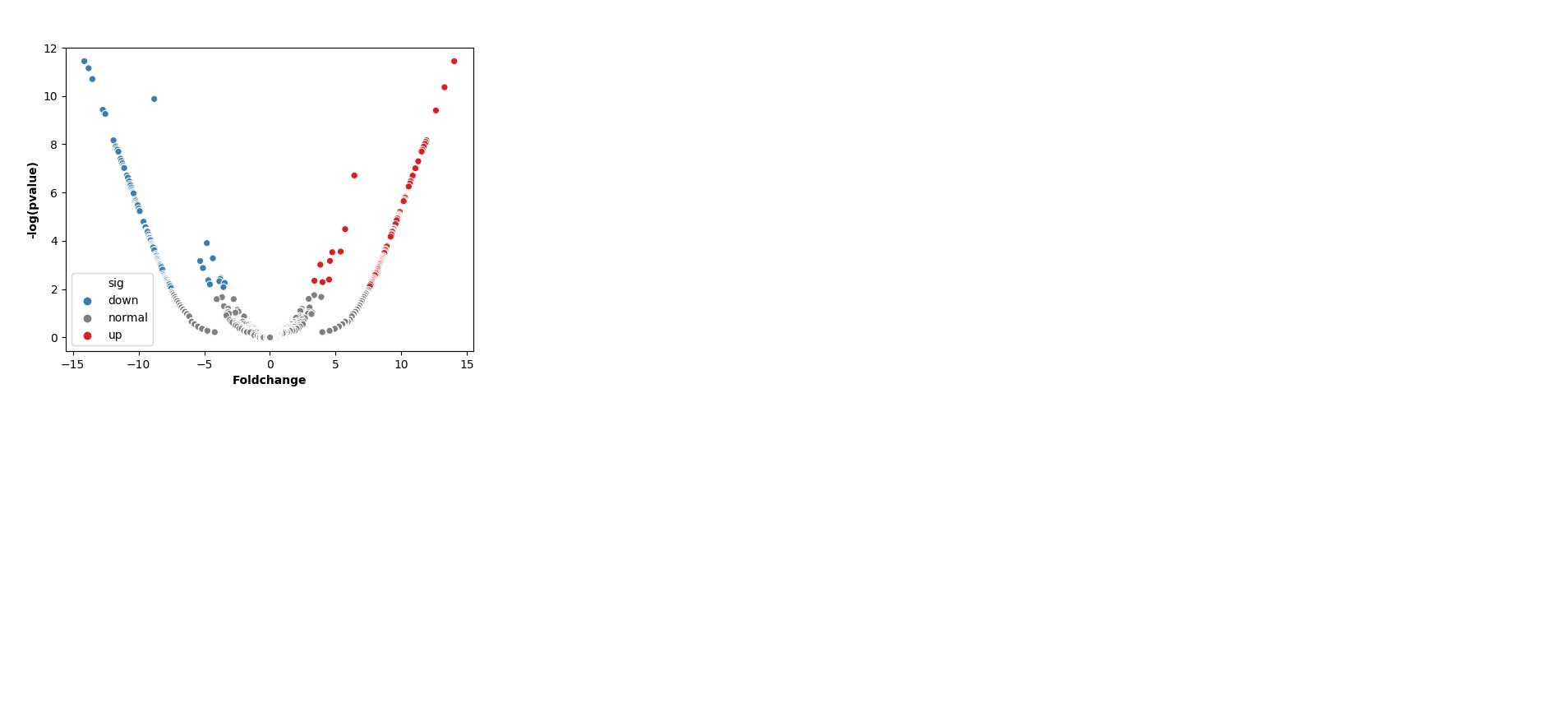

Supplement: FIGURE S2 — Visualization of differentially expressed circRNAs induced by SUS. [file Image_2.JPEG]

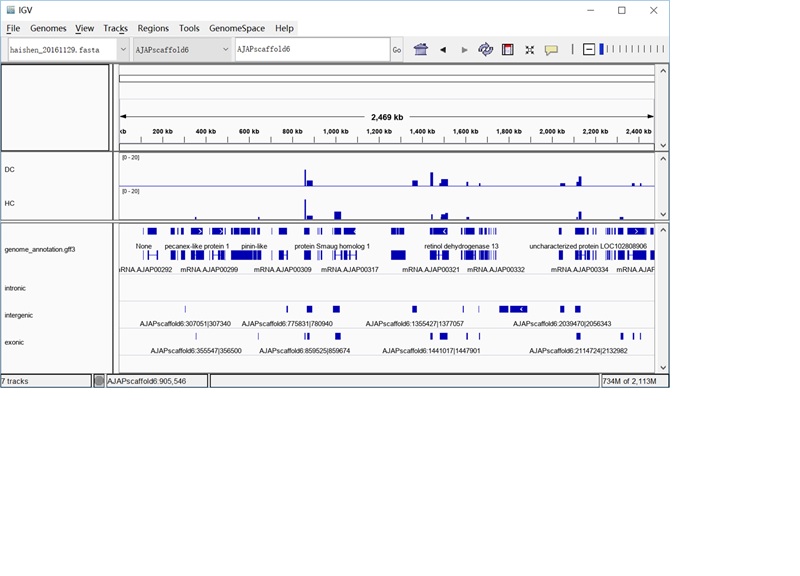

Supplement: FIGURE S3 — Example of visualization of circRNAs on the genome by IGV. DC, HC: the expression of circRNAs in each group. genome_annotation.gff3: the annotation file of sea cucumber genome. [file Image_3.JPEG]
