# Supplementary material for: Genome-Wide Identification of Circular RNAs Revealed the Dominant Intergenic Region Circularization Model in Apostichopus japonicus
Source: Front Genet. 2019 Jul 2;10:603. doi: 10.3389/fgene.2019.00603 (PMC6614181; doi:10.3389/fgene.2019.00603)
Supplement: TABLE S1 — Primers for validation of circRNAs. [file Table_1.DOCX]

**Table S1 primers for validation of circRNAs**

| circRNAs | circRNAs type | Primers (5’-3’) | Validation experiments |
| --- | --- | --- | --- |
| AJAPscaffold1004:153890\|154913 | Exon-exon | F: GAGGAAGTTCAAGGCAAGAC | Used for Sanger sequencing and qRT-PCR |
|  |  | R: AAATCCTGCGTGGAGAAG |  |
| AJAPscaffold763:53699\|54973 | Exon-exon | F: GCAAACAGGGATTCAACG | Used for Sanger sequencing and qRT-PCR |
|  |  | R: GCTGATTTCTTCGGATGG |  |
| AJAPscaffold1149:136110\|200955 | Exon-intron | F: CTTCCGTCTGGATGGTCG | Used for Sanger sequencing, qRT-PCR and the RNase R treated experiment |
|  |  | R: GGTGTCCCGTCGGAGGTAG |  |
| AJAPscaffold254:100722\|267769 | Exon-intron | F: GCACGGGAGGAGCAAGAT | Used for Sanger sequencing and qRT-PCR |
|  |  | R: GCATCGTTATGGCACTCA |  |
| AJAPscaffold1365:65949\|74883 | Intron | F: TTCGCTGGAGATGATGCT | Used only for Sanger sequencing |
|  |  | R: TGTGACGATACCGTGTTC |  |
| AJAPscaffold631:79430\|101056 | Intron | F: CAGTAAACTTCCGATAGA | Used only for Sanger sequencing |
|  |  | R: CAAACACCTTCACCACCG |  |
| AJAPscaffold65:385508\|395157 | Intergenic | F: GACGGTAGACTTACAGAA | Used only for Sanger sequencing |
|  |  | R: AACTCAACGGAGAAACTA |  |
| AJAPscaffold880:116912\|124478 | Intergenic | F: GCCGCAGATAATCAGAAATA | Used for Sanger sequencing and qRT-PCR |
|  |  | R: CAACCCATTTCGTTATTC |  |
| AJAPscaffold114:640549\|706803 | Exon-intron | F: CTAGCCGTTAGTAGGTGAA | Used only for qRT-PCR |
|  |  | R: AGGCATCACTTGTTCCAG |  |
| AJAPscaffold194:80613\|138777 | Exon | F: CACAGACAGTTCTGGTGG | Used only for qRT-PCR |
|  |  | R: AATGCGGAAGTTATCGTA |  |
| β-actin | Linear mRNA | F: CCATTCAACCCTAAAGCCAACA | Used as a reference for qRT-PCR |
|  |  | R: ACACACCGTCTCCTGAGTCCAT |  |
| Ficolin | Linear mRNA | F: AACGTTCCGTCGTCAGATCAAAC | Used for the RNase R treated experiment, as the host gene of AJAPscaffold1149:136110\|200955 |
|  |  | R: TCATGAAGAAGGTCGAATTTTCA |  |
