# Supplementary material for: Genome-Wide Identification of Circular RNAs Revealed the Dominant Intergenic Region Circularization Model in Apostichopus japonicus
Source: Front Genet. 2019 Jul 2;10:603. doi: 10.3389/fgene.2019.00603 (PMC6614181; doi:10.3389/fgene.2019.00603)
Supplement: TABLE S2 — Statistics of the quality of circ-seq data. [file Table_2.DOCX]

**Table S2 Statistics of the quality of the circ-seq data**

| Sample name | Raw reads | Clean reads | Clean bases | Error rate (%) | Q20 (%) | Q30 (%) | GC content (%) |
| --- | --- | --- | --- | --- | --- | --- | --- |
| DC | 211,004,636 | 207,620,356 | 31.14 G | 0.02 | 97.65 | 96.16 | 42.21 |
| HC | 236,349,396 | 231,127,910 | 34.67 G | 0.02 | 97.69 | 96.22 | 43.18 |
